# Automated semantic annotation of rare disease cases: a case study

**DOI:** 10.1093/database/bav107

**Published:** 2015-11-13

**Authors:** Maria Taboada, Hadriana Rodríguez, Diego Martínez, María Pardo, María Jesús Sobrido

doi:10.1093/database/bau045

The authors of the earlier article would like to include the below funding statement for this article.

## Funding

Instituto de Salud Carlos III (grant no. FIS2012-PI12/00373:OntoNeurophen). Funding for open access charge: Instituto de Salud Carlos III (grant no. FIS2012-PI12/00373) and FEDER (European funding).

